# Bioadsorbents from Household Biowastes: A Sustainable Solution for CO_2_ Capture

**DOI:** 10.3390/ma19101937

**Published:** 2026-05-08

**Authors:** Marcelina Sołtysik, Izabela Majchrzak-Kucęba, Dariusz Wawrzyńczak

**Affiliations:** Department of Advanced Energy Technologies, Faculty of Infrastructure and Environment, Czestochowa University of Technology, Dabrowskiego Street 73, 42-201 Czestochowa, Poland; izabela.majchrzak-kuceba@pcz.pl (I.M.-K.); dariusz.wawrzynczak@pcz.pl (D.W.)

**Keywords:** household biowastes, biocarbon, bioadsorbent, chemical activation, KOH, activated carbon, adsorption, CO_2_ capture, flue gas

## Abstract

Bioadsorbents derived from food waste can not only help reduce the amount of such waste but also demonstrate significant potential for CO_2_ capture from both the energy sector and other industries. This study evaluates the feasibility of using bioadsorbents obtained from various types of household biowaste—including black and green coffee grounds, tea grounds, potato peels, walnut shells and green walnut shells—for CO_2_ capture from flue gas. The bioadsorbents were produced through a two-step process consisting of carbonization followed by KOH activation. The physicochemical properties of the bioadsorbents were characterized using SEM, FTIR, XRD, TGA and BET techniques. The CO_2_ sorption capacity was examined for bioadsorbents and for the original biowaste and the biocarbons obtained after carbonization. Isothermal CO_2_ adsorption tests were carried out at 25 °C under 100% CO_2_ atmosphere. The influence of porous properties—such as specific surface area, total pore volume, micropore volume and average pore diameter—on the CO_2_ sorption capacity was assessed for bioadsorbents, biocarbons and raw biowastes. The results showed that the most effective bioadsorbent for CO_2_ capture was derived from spent dark roast coffee grounds, with a sorption capacity of 115.8 mg_CO2_/g_A_. The favorable sorption performance of this bioadsorbent was attributed to its high specific surface area (1580 m^2^/g), the largest total pore volume (0.84 cm^3^/g) and micropore volume (0.5 cm^3^/g) among the tested materials, as well as an optimal average pore diameter (0.96 nm). Similarly favorable structural properties were observed for the potato peel-derived bioadsorbent (APP—1604 m^2^/g; 0.65 cm^3^/g) and the bioadsorbent derived from green walnut shells (AGWS—1376 m^2^/g; 0.64 cm^3^/g). Their CO_2_ adsorption capacities reached 104.1 mg_CO2_/g_A_ and 73.2 mg_CO2_/g_A_, respectively, for AGWS and APP.

## 1. Introduction

Reduction in greenhouse gas emissions, particularly carbon dioxide (CO_2_), alongside the transition to a circular economy, is a key measure in mitigating climate change. Proposed solutions in this area include improving energy generation efficiency, transitioning to renewable energy sources, and implementing carbon capture, utilization, and storage (CCUS) technologies [1,2]. For post-combustion CO_2_ capture, absorption methods using liquid solvents and adsorption methods employing solid sorbents—such as activated carbons or zeolites—are primarily considered [3]. The use of solid physical adsorbents offers advantages due to their ease of regeneration and the resulting lower energy consumption of the overall process. The development of adsorption-based CO_2_ capture technologies, such as Vacuum Pressure Swing Adsorption (VPSA), Pressure Swing Adsorption (PSA), and Temperature Swing Adsorption (TSA), is being advanced both through process cycle optimization and the testing of novel adsorbent materials, which may significantly enhance CO_2_ capture efficiency [4,5].

Among the emerging and promising sorbent materials [6,7,8] are bioadsorbents derived from biowaste [9,10,11,12,13,14,15], including food waste [16,17,18,19,20,21,22,23]. In the context of increasing waste generation and the need for effective waste management, the valorization of biowaste is gaining growing importance as a component of the circular economy. Biowaste includes biodegradable materials from gardens and parks, as well as food waste originating from households, food services, and food processing industries. These materials constitute a significant portion of total municipal solid waste [24]. A wide variety of biowaste is generated on a large scale, particularly in the food and agricultural sectors. Globally, the largest volumes of biowaste are generated from the processing of sugarcane (400–600 million tons per year), rice straw (approximately 490 million tons per year), potatoes (54 million tons per year), coffee (6.5 million tons per year), and tea (5.4 million tons per year) [25,26,27]. Walnut processing waste also constitutes a considerable share. While potato processing waste is often utilized in biogas plants, potato peels, especially those generated in households, remain a challenge [28]. Walnut shells, on the other hand, are mainly used as biofuel due to their high calorific value [29]. Coffee grounds are primarily disposed of in landfills, but they are also used as fuel in industrial boilers due to their high calorific value, or as a component in compost production. In recent years, spent coffee grounds have attracted increasing interest as a resource for the production of bioactive phenolic compounds, biofuels, biochar-based catalysts, and CO_2_ bioadsorbents [17,30,31]. In turn, small quantities of tea grounds are utilized by companies for caffeine extraction, while decaffeinated tea residues are used as poultry feed [32]. Thus, biowaste does not necessarily have to end up in landfills—it can also be converted into valuable products such as compost, biogas, or bioadsorbents [33,34].

Bioadsorbents are carbonaceous materials that, due to their inherent hydrophobicity, high nitrogen content, and thermal stability, possess significant potential for applications such as hydrogen storage, heavy metal removal from water, and purification of exhaust gases, including carbon dioxide capture from boiler flue gases [19,35,36,37]. The literature offers numerous examples of the use of bioadsorbents as alternatives to traditional carbon dioxide adsorbents [38,39,40,41,42,43]. Their main advantages, beyond high specific surface area and microporous structure, include the following: low production cost resulting from the utilization of biowaste (biomass, solid waste, food waste); hydrophobicity, which ensures moisture resistance and operational stability under harsh industrial conditions; and biodegradability and environmental friendliness, making them a sustainable solution. Bioadsorbents can be produced from biowaste through various methods [38,44]. One commonly applied method is a two-step process consisting of carbonization followed by chemical or physical activation. Carbonization involves the thermal decomposition of biowaste under oxygen-limited conditions, leading to the formation of biochars with high carbon content [38]. Activation—either chemical or physical—further develops the porous structure, significantly increasing surface area and enhancing the material’s sorption capacity. Among activation methods, chemical activation using potassium hydroxide (KOH) has shown particular promise in promoting microporosity and, consequently, enhancing CO_2_ adsorption performance [45,46,47,48]. Bioadsorbents derived from biowaste via carbonization and KOH chemical activation, as reported in the literature [16,36,38,39,49,50], exhibit specific surface areas ranging from 587 m^2^/g (MZn48FAL), derived from African palm biomass [51], to 2695 m^2^/g (PC3-780), derived from rice husks [52] and cotton stalk bioadsorbents [53].

However, KOH activation has certain drawbacks, such as high chemical consumption and environmental concerns. Therefore, in recent years, alternative activation methods have been increasingly explored. In particular, molten salt pyrolysis has emerged as a promising approach, enabling the simultaneous carbonization and structural tuning of biomass in an ionic medium. This method allows for better control over pore structure development, including micro- and mesoporosity, and can improve the structural stability of the resulting carbon materials. Furthermore, the type of salt and processing conditions significantly influence defect formation and adsorption properties, making this approach a versatile alternative to conventional chemical activation [54,55].

According to the available data [16,36,38,39,40,49,50,51], bioadsorbents proposed for CO_2_ capture from flue gases have primarily been obtained from spent coffee grounds [16,38,39,40], walnut shells [31,49,50,51], African palm biomass [56,57,58], rice husks [52,59,60], date seeds [41,42,43,44], as well as from pomegranate peels [22], carrot peels [61], fern leaves [61], and cotton stalks [53]. The CO_2_ adsorption capacities of these bioadsorbents, measured at 298 K and 1 bar, ranged from 95.5 mg_CO2_/g_A_ (date seed-based AC-2.5-600) [44] to 238 mg_CO2_/g_A_ (date seed-based HTC-PDS_KOH_1) [43]. High sorption capacities were also recorded for coconut shell-derived NC-650-4 (210 mg_CO2_/g_A_) [31], spent coffee ground-derived CG800-1 and CG700-2-1 (195 and 193.5 mg_CO2_/g_A_, respectively) [38,39], carrot peel-derived bioadsorbents (185 mg_CO2_/g_A_) [61], and cotton stalk-derived materials (186 mg_CO2_/g_A_) [53]. These high CO_2_ uptake values indicate the substantial potential of biowaste-derived bioadsorbents for CO_2_ capture in adsorption-based installations [16,38,39,49,50,51,52]. The aim of this study was to evaluate the potential of a specific group of biowaste—namely household biowastes—for the production of effective CO_2_ bioadsorbents. As current research shows [16,38,39,49,50,51,52], both the selection of biowaste precursors and the activation method are crucial for obtaining carbonaceous materials with porous properties suitable for effective CO_2_ sorption. Therefore, it would be particularly valuable to investigate biowaste sources not yet explored for CO_2_ bioadsorbent production, such as green coffee grounds, green walnut husks and potato peels. Such research was conducted as part of this study. This may allow for the expansion of the range of biowastes that can be used to obtain effective CO_2_ adsorbents in the future. The existing studies typically focus on the modification of a single type of biowaste. The novelty of this article lies in presenting, for the first time, a systematic investigation involving the modification of six different types of household biowastes—including three types not previously tested for this purpose—under identical process conditions. Notably, there is a lack of studies examining CO_2_ sorption capacity not only on different types of bioadsorbents but also on their precursors—that is, the biowastes (household biowastes) and the biochars obtained from them. Such research was conducted in this work. Moreover, comprehensive analyses that would evaluate how the transformation of porous structures during carbonization and subsequent activation impacts the CO_2_ adsorption performance of these materials are missing. Researchers have primarily focused on investigating the porous structure and CO_2_ adsorption capacity of already synthesized bioadsorbents, without addressing the corresponding properties of the intermediate products (biochars obtained after carbonization) and the original precursors, i.e., the biowastes. This article presents, for the first time, a comprehensive analysis of the relationship between the porous structure of the resulting bioadsorbents and their precursors (biochars and biowastes), as well as their CO_2_ adsorption capacities. This approach has yielded valuable insights into the transformation process of household biowastes into efficient carbon dioxide bioadsorbents.

## 2. Materials and Methods

### 2.1. Preparation of Bioadsorbents

**Figure 1:**
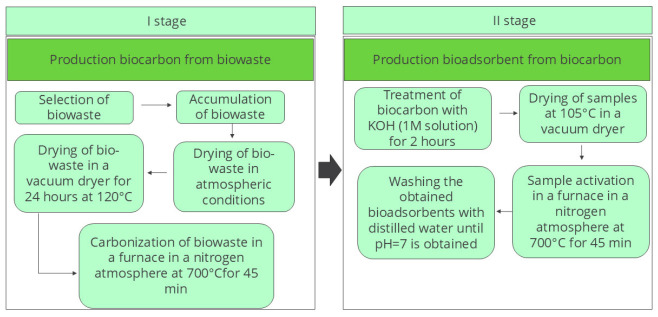
Scheme of the bioadsorbent preparation from the biowaste.

Six different types of household biowastes were generated in significant quantities worldwide, particularly in domestic environments, and thus characterized by high availability; they were used for the preparation of bioadsorbents. These were as follows: (1) spent grounds from brewing high roasted coffee beans (high roasted coffee grounds—HRoCG), (2) potato peelings (potato peelings—PP), (3) spent tea grounds (tea grounds—TG), (4) green walnut shells (green walnut shells—GWS), (5) walnut shells (walnut shells—WS), (6) spent grounds from brewing green coffee beans (green coffee grounds—GCG). A detailed analysis of the biowastes and biochars was presented in the authors’ earlier work [11]. The bioadsorbents were prepared through a two-step process: (I) carbonization of the biowastes and (II) chemical activation of the resulting biochars using a KOH solution. The biochars obtained after carbonization were denoted with the prefix “C” (CHRoCG, CPP, CTG, CGWS, CWS, CGCG) and the final bioadsorbents obtained after activation were denoted with the prefix “A” (AHRoCG, APP, ATG, AGWS, AWS, AGCG). Details of the procedure are presented in Figure 1. Additional information can be found in the authors’ publication [11].

In stage (I), the biowastes were initially air-dried at room temperature and subsequently dried in a vacuum oven at 120 °C for 24 h, which allowed for moisture removal and preparation of the biowastes for the carbonization process. Carbonization was carried out under a nitrogen atmosphere at 700 °C for 45 min. The nitrogen flow prevented oxidation, while the high temperature enabled the decomposition of organic matter, leading to the formation of porous carbon structures characteristic of biochars. Stage (II) involved the chemical activation of the biochars using a KOH (Chempur, Piekary Śląskie, Poland, laboratory quality) solution for 2 h (KOH: biomass—2:1). Following this step, the samples were dried in a vacuum oven at 105 °C and then thermally activated under a nitrogen atmosphere at 700 °C for 45 min. In the final step, the bioadsorbents were washed with distilled water until a neutral pH (pH = 7) was reached, in order to remove residual KOH.

### 2.2. Characterization of the Bioadsorbents

The physicochemical properties of the bioadsorbents were characterized by SEM, FTIR, XRD, TG and nitrogen adsorption/desorption BET analysis. The morphological features of the bioadsorbents were investigated by scanning electron microscopy (SEM, Hitachi S-3400N, Tokyo, Japan). The microscope, equipped with a secondary electron (SE) detector and a backscattered electron (BSE) detector, enabled the acquisition of high-resolution images. The samples were scanned at voltages ranging from 5 kV to 20 kV, depending on the requirements of the specific analysis. The Fourier transform infrared spectra for the samples were measured at room temperature on a Nicolet iS10 spectrometer (Thermo Fisher Scientific, Waltham, MA, USA), in the range of 4000–400 cm^−1^ using the KBr pellet technique. To prepare the pellet, 0.001 g of the sample and 0.2 g of KBr were used. The phase composition of the samples was determined by powder X-ray diffraction (P-XRD) using a Philips X’Pert instrument (Philips, Amsterdam, The Netherlands) equipped with an X’Celerator Scientific detector. Diffraction data were recorded between 5° and 60° 2θ at an interval of 0.08° 2θ. The thermal properties of the bioadsorbents were characterized by thermogravimetric analysis (TG) on a Mettler TGA/DSC1 thermobalance (Mettler–Toledo, Greifensee, Switzerland). About 4 mg of the bioadsorbent was heated at 10 °C min^−1^ from 25 °C to 800 °C in a nitrogen flow (100 mL min^−1^). The porosity characteristics of the samples were determined by N_2_ adsorption–desorption isotherms performed at −196 °C on a Micromeritics ASAP 2010 analyzer (Micromeritics, Norcross, GA, USA). The specific surface area was calculated by the BET method from the linear part of the BET plot according to IUPAC recommendations using the adsorption isotherm (relative pressure (*p*/*p*_0_) = 0.05–0.23). The total pore volume (V_p_) was determined based on the maximum amount of N_2_ adsorbed within the pores of the samples at *p*/*p*_0_ = 0.99. The pore size distribution was calculated using the DFT method. The micropore volume (W_o_) was determined based on the N_2_ adsorption isotherm using the Dubinin–Radushkevich (DR) equation, assuming an adsorbed phase density of 0.808 cm^3^ g^−1^ and a cross-sectional area of 0.162 nm^2^ [62]. The average pore width (L_o_) was calculated using the Stoeckli–Ballerini equation [63]. The samples were degassed at 300 °C overnight on a high vacuum line prior to adsorption.

### 2.3. CO_2_ Capture

**Figure 2:**
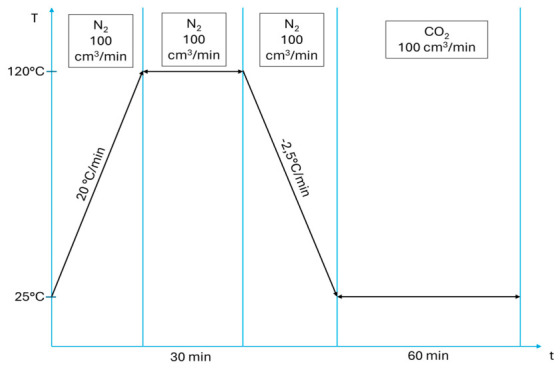
Isothermal adsorption test (IAT).

The examination of CO_2_ sorption capacity was carried out using a Mettler TGA/DSC1 thermobalance. The studies were performed for six bioadsorbents and their precursors (biowastes, biocarbons) using the isothermal adsorption test (IAT), the course of which is shown in Figure 2.

The proposed CO_2_ thermogravimetric test utilizes the isothermal adsorption test (IAT). In the isothermal test, samples of bioadsorbents, biocarbons and biowastes were heated from 25 °C to 120 °C in a nitrogen atmosphere (with a heating rate of 20 °C min^−1^) and held at this temperature for 30 min (until a constant sample mass was achieved). The sample was then cooled to 25 °C under a nitrogen atmosphere (at a heating rate of 20 °C min^−1^), at which the CO_2_ adsorption process was carried out. Then the CO_2_ sorption process was conducted isothermally at a temperature of 25 °C and being held at that temperature until an equilibrium state was attained, under atmospheric pressure, using 100% (more specifically, 99.999%) CO_2_ at a flow rate of 100 cm^3^ min^−1^.

## 3. Results and Discussion

### 3.1. Physicochemical Properties of the Adsorbents

**Figure 3:**
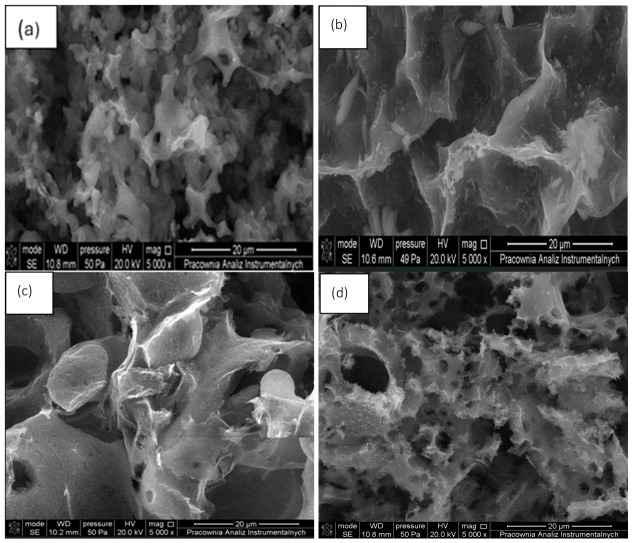

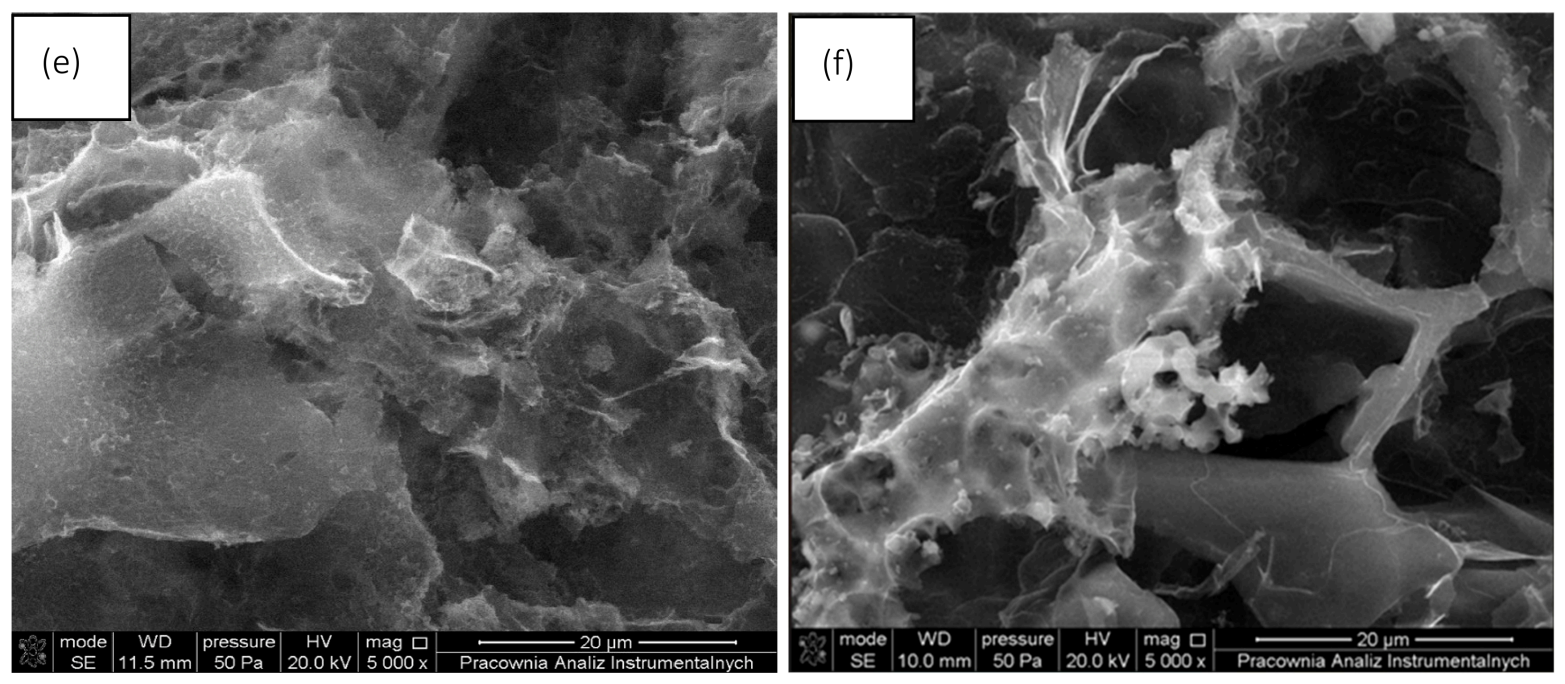
SEM of bioadsorbents: (**a**) AHRoCG, (**b**) APP, (**c**) ATG, (**d**) AGWS, (**e**) AWS, and (**f**) AGCG.

The structure and morphology of the obtained bioadsorbents (AHRoCG, APP, ATG, AGWS, AWS, AGCG) are presented in Figure 3.

**Figure 4:**
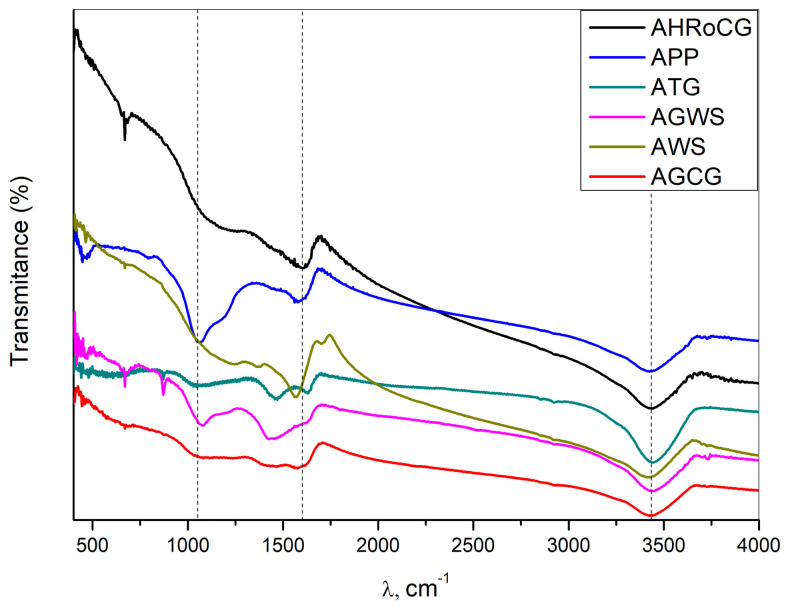
FTIR profiles for bioadsorbents: AHRoCG, APP, ATG, AGWS, AWS, AGCG.

SEM images (Figure 3) of all the investigated bioadsorbents indicate a significant porosity of the obtained materials. In the case of the adsorbents derived from coffee grounds (AHRoCG—Figure 3a), potato peels (APP—Figure 3b) and green walnut shells (AGWS—Figure 3d), a high density of micropores is observed, confirming a substantial microporous structure in comparison to the remaining samples. Particularly for the adsorbent prepared from high roasted coffee grounds (AHRoCG—Figure 3a), a highly regular network of fine micropores is noticeable. The structure of the adsorbent from walnut shells (AGWS—Figure 3d) is also noteworthy, consisting of both numerous micropores and macropores. FTIR spectra of the obtained bioadsorbents are presented in Figure 4.

**Figure 5:**
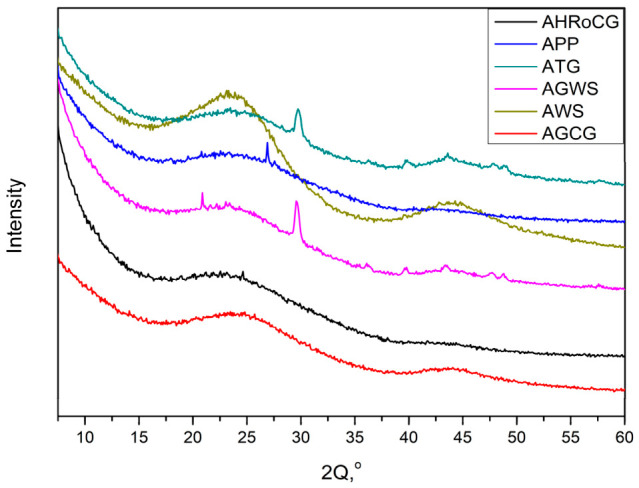
XRD of bioadsorbents: AHRoCG, APP, ATG, AGWS, AWS, AGCG.

**Figure 6:**
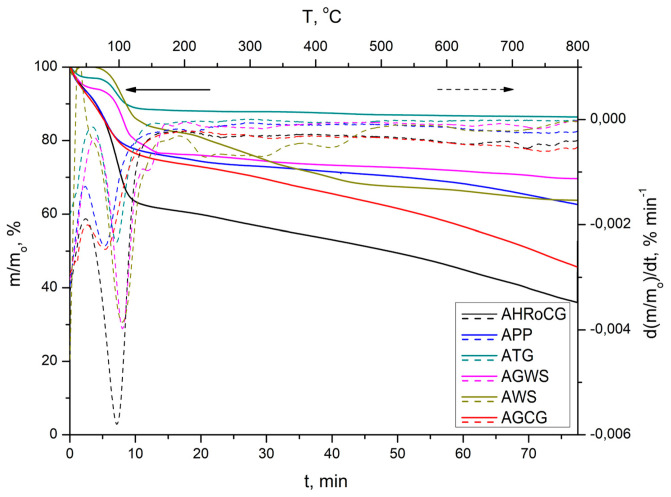
TG and DTG curves of bioadsorbents: AHRoCG, APP, ATG, AGWS, AWS, AGCG.

The FTIR spectra of all the bioadsorbents show characteristic absorption bands corresponding to stretching vibrations of C=O in carboxylic groups (at 1720 cm^−1^) and hydroxyl groups of –OH present on the surface of the tested materials (at 3400 cm^−1^) [64]. The XRD diffractograms of the obtained bioadsorbents are shown in Figure 5. As seen in Figure 5, the spectra of the bioadsorbent samples are similar and exhibit a smooth profile, which indicates a low degree of crystallinity. Sharp peaks at 2θ = 27°, 30°, and 44°, visible in the diffractograms of AGWS, ATG, and APP bioadsorbents, are assigned to disordered graphitic planes [64,65]. The TG and DTG curves of the bioadsorbents are presented in Figure 6.

Figure 6 presents the profiles of thermal decomposition (TG curve) and their derivatives (DTG curve) of bioadsorbents during heating in an inert atmosphere (N_2_) and the rate of 10 °C min^−1^.

**Figure 7:**
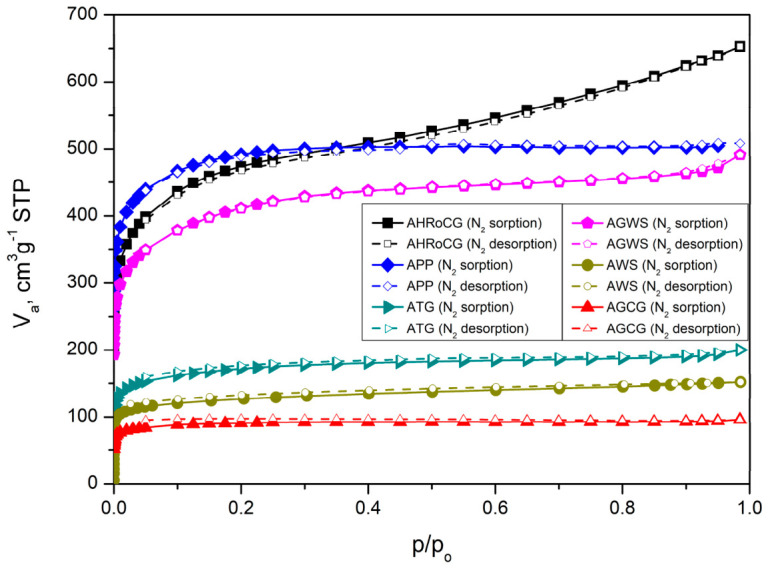
Nitrogen adsorption isotherms for the bioadsorbents: AHRoCG, APP, ATG, AGWS, AWS, and AGCG.

As shown by the TG curves (Figure 6), the total mass loss of all bioadsorbents varied and ranged from 15% for AHRoCG to 63% for ATG. In the case of bioadsorbents, one significant mass loss (25–150 °C) can be observed, related to dehydration. The second mass loss (220–550 °C) amounts to about 20% for AHRoCG, AWS, and AGCG, and results from the thermochemical conversion of biopolymer fractions. This process includes the decomposition of cellulose and hemicellulose (with the maximum peak observed on the DTG curve at 300 °C), as well as probable lignin degradation (around 400 °C) [66]. Figure 7 presents the N_2_ adsorption and desorption isotherms for the obtained bioadsorbents.

**Figure 8:**
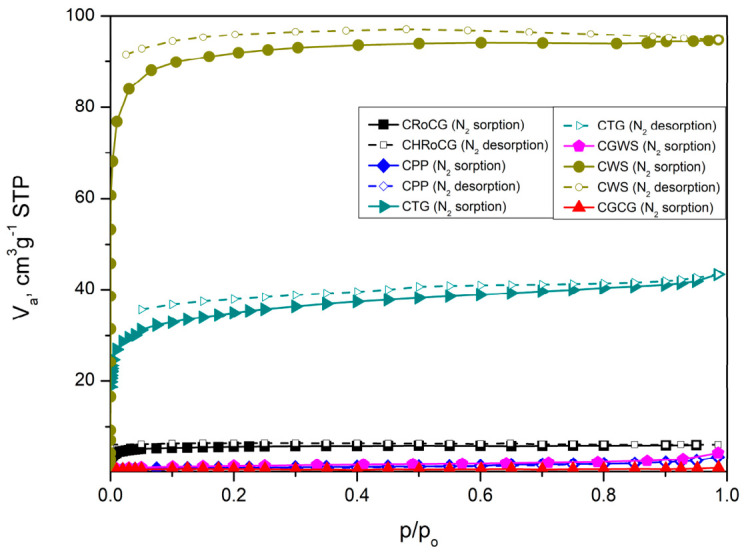
The isotherms of biocarbons: CHRoCG, CPP, CTG, CGWS, CWS, CGCG.

The isotherms of the bioadsorbents (Figure 7) (AHRoCG, APP, ATG, AGWS, AWS, AGCG) correspond to type I isotherms according to the International Union of Pure and Applied Chemistry (IUPAC) classification, indicating that most of the nitrogen is adsorbed at low relative pressures without the presence of a hysteresis loop. A type I isotherm suggests that the adsorbent is predominantly microporous [67]. This confirms the development of a microporous structure during the KOH activation process of the biocarbons. In contrast, the isotherms obtained for the biocarbons (Figure 8) are predominantly type IV, except for the biocarbons derived from green walnut shells (CGWSs) and green coffee grounds (CGCGs). This type of isotherm is characteristic of mesoporous materials. The biocarbons exhibit H2-type hysteresis loops, which are indicative of open pores with significant constrictions. A low pore volume and the presence of H4-type hysteresis indicate the existence of narrow slit-shaped pores formed between parallel planes.

**Figure 9:**
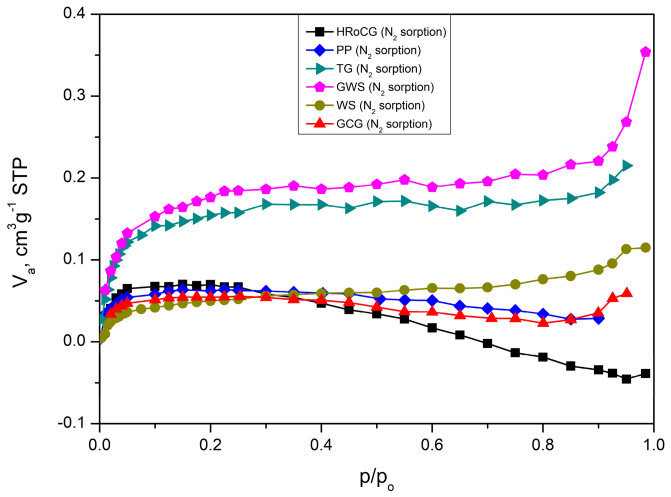
The isotherms of biowastes: HRoCG, PP, TG, GWS, WS, GCG.

In contrast, the raw biowastes (Figure 9) exhibit type VI isotherms. This type of flat, linear isotherm is characteristic of homogeneous materials with a very poorly developed porous structure.

**Figure 10:**
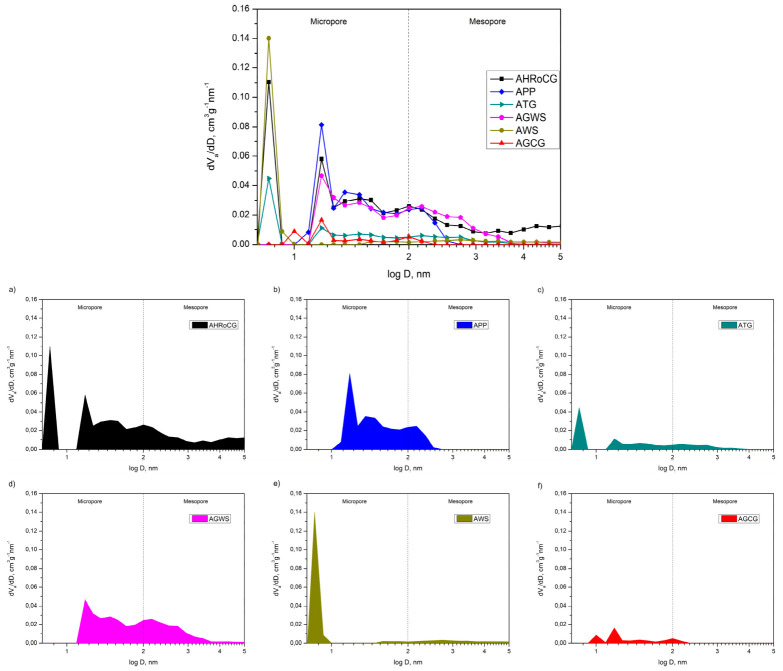
The pore volume distribution curves according to pore diameter for the bioadsorbents: (**a**) AHRoCG; (**b**) APP; (**c**) ATG; (**d**) AGWS; (**e**) AWS; (**f**) AGCG.

Figure 10 presents the pore volume distribution curves according to pore diameter for the bioadsorbents. For the bioadsorbent samples (Figure 10), the largest contribution to the total pore volume corresponds to pores with diameters of 0.86 nm (for AHRoCG), 1.18 nm (for APP), 0.86 nm (for ATG), 1.18 nm (for AGWS), 0.86 nm (for AWS), and 1.18 nm (for AGCG). This confirms that the obtained bioadsorbents possess a microporous structure, dominated by pores with diameters below 1.18 nm, although the presence of mesopores is also observed.

**Figure 11:**
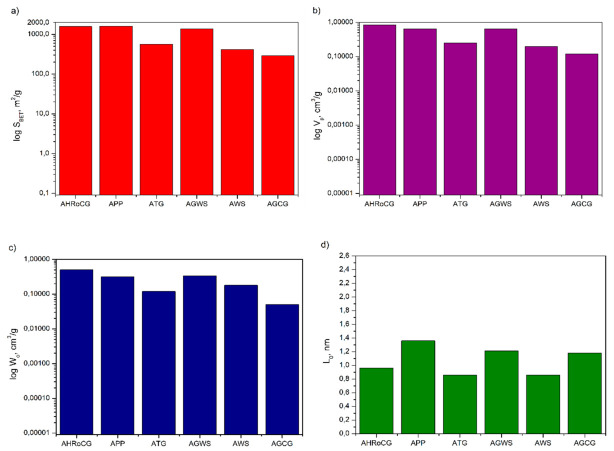
Surface area (**a**), pore volume (**b**), micropore volume (**c**) and pore diameter (**d**) of bioadsorbents.

**Figure 12:**
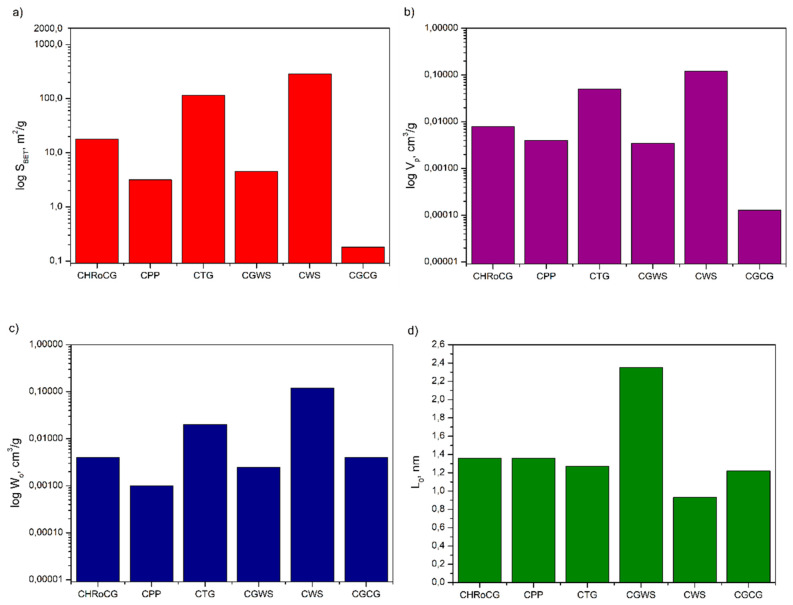
Surface area (**a**), pore volume (**b**), micropore volume (**c**) and pore diameter (**d**) of biocarbons.

**Figure 13:**
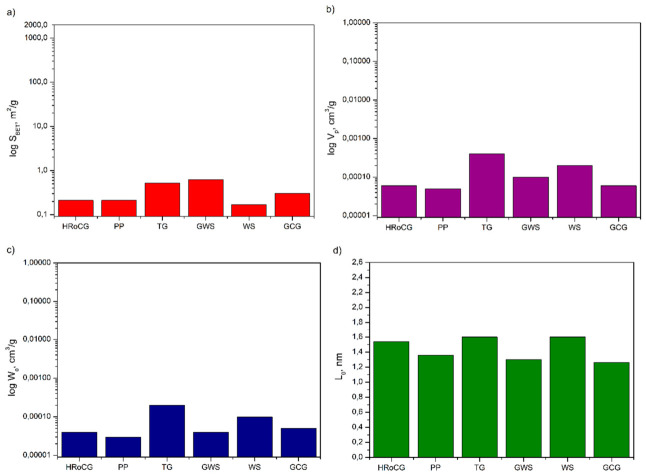
Surface area (**a**), pore volume (**b**), micropore volume (**c**) and pore diameter (**d**) of food biowastes.

In Figure 11, Figure 12 and Figure 13, the pore structure parameters such as surface area, pore volume, micropore volume and pore diameter for the bioadsorbents, as well as for comparison for biocarbons and biowastes, are gathered. As shown in Figure 11, Figure 12 and Figure 13, the carbonization of biowastes, particularly the activation of biocarbons, significantly affected the changes in the pore structure parameters of the studied materials. The specific surface area of the initial biowastes selected for the study was modest, ranging from 0.04 m^2^/g (for GCG) to 0.62 m^2^/g (for GWS) (Figure 13a). The biocarbons obtained through carbonization already had a higher specific surface area, ranging from 0.18 m^2^/g (for CGCG) to 289 m^2^/g (for CWS) (Figure 12a). A significant increase in the specific surface area was observed for the obtained bioadsorbents (Figure 11a). The highest specific surface areas were found for the bioadsorbent made from potato peelings (APPs) (1604 m^2^/g), the bioadsorbent from highly roasted coffee grounds (AHRoCG) (1580 m^2^/g) and the bioadsorbent from green walnut shells (AWGSs) (1376 m^2^/g). The remaining bioadsorbents had specific surface areas ranging from 293 m^2^/g (for AGCG) to 564 m^2^/g (for ATG). The three bioadsorbents with the largest specific surface areas (S_BET_), i.e., the bioadsorbent from potato peelings (APPs), the bioadsorbent from highly roasted coffee grounds (AHRoCG) and the bioadsorbent from green walnut shells (AGWSs), also exhibited the largest pore volumes (V_p_) of 0.65 cm^3^/g, 0.84 cm^3^/g, and 0.64 cm^3^/g, respectively (Figure 11b), as well as the highest micropore volumes (W_0_) of 0.32 cm^3^/g, 0.5 cm^3^/g, and 0.34 cm^3^/g, respectively (Figure 11c).

The bioadsorbent from highly roasted coffee grounds (AHRoCG) exhibited the largest pore volume (0.84 cm^3^/g) and the highest micropore volume (0.5 cm^3^/g). Additionally, the pores of this bioadsorbent had an average pore diameter (L_0_) of 0.96 nm (Figure 11d). This indicates the highly favorable pore structure properties of this bioadsorbent, with a well-developed microporosity, which is particularly important for CO_2_ adsorption. The smallest average pore diameter (L_0_) of 0.86 nm was observed for the bioadsorbent made from tea grounds (ATGs) and the bioadsorbent from walnut shells (AWSs), while the largest average pore diameter was found for the bioadsorbent from potato peelings (APPs) at 1.36 nm. These results confirm that all bioadsorbents exhibited a developed pore structure with numerous micropores. A minor degree of microporosity was noted in a few of the biocarbons (CWS, CTG) (Figure 12). The structure of all biowastes, on the other hand, contained a minimal amount of micropores, often at the detection limit of the analysis (Figure 13).

This confirms that the initial biowastes, as precursors to bioadsorbents, have a poorly developed porous surface. As shown in Figure 11, Figure 12 and Figure 13, the processes of biowaste carbonization followed by chemical activation with KOH of the resulting biocarbons proved to be effective and crucial for obtaining bioadsorbents with a porous structure, numerous micropores, large pore volume, and a high specific surface area. In summary, although the activation method with KOH resulted in varying outcomes for the studied biocarbons, it influenced the type of porous structure of the obtained bioadsorbents. The applied KOH showed distinct activation effects, as potassium (K) easily penetrates the aromatic structure of the biocarbon, shaping its porous structure and surface activity, leading to the creation of an effective bioadsorbent—porous carbon.

The chemical activation with KOH, resulting in the formation of porosity, involves a solid–liquid reaction, including metal oxide reduction and carbon oxidation reactions [68]. Since potassium metal is highly active and mobile at the activation temperature, it is easy to introduce it into the carbon matrix. As a result, the layered graphite structure expands, forming pore structures. Thus, the high specific surface area obtained through KOH activation can be attributed to the insertion of potassium (K) [69]. Additionally, the applied KOH solution activates carbon materials (biocarbons), creating a more microporous structure without promoting the formation of meso- and macropores.

### 3.2. CO_2_ Capture by Biowaste, Biocarbon and Bioadsorbents

**Figure 14:**
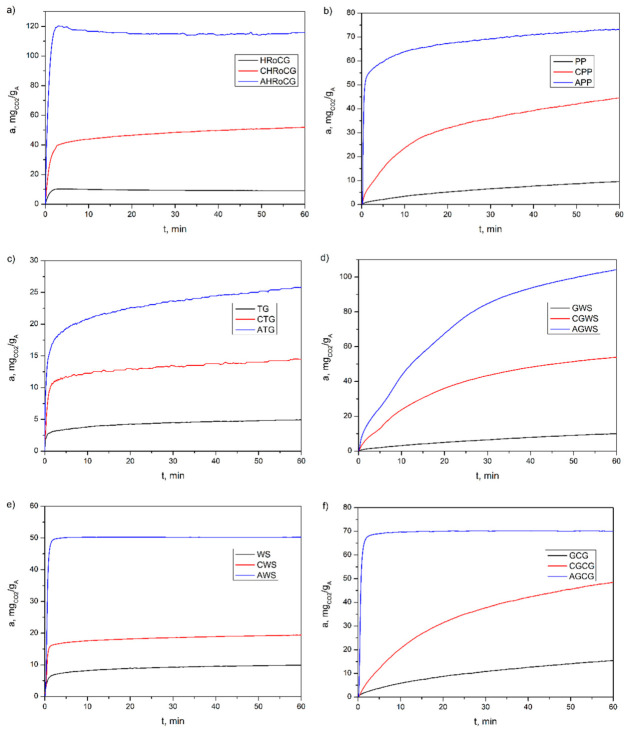
The CO_2_ adsorption profiles for biowastes, biocarbons and bioadsorbents coming from six different base materials: (**a**) high roasted coffee grounds (HRoCG), (**b**) potato peelings (PPs), (**c**) tea grounds (TGs), (**d**) green walnut shells (GWSs), (**e**) walnut shells (WSs) and (**f**) green coffee grounds (GCGs).

The CO_2_ adsorption profiles (Figure 14) obtained for the modified biocarbons (red line) and bioadsorbents (blue line) showed very fast adsorption, allowing nearly 100% of the equilibrium value to be reached within the first 3 min of the adsorption process, for the bioadsorbents AHRoCG, AWS, and AGCG. A similar characteristic is also observed for the bioadsorbents APP and ATG, although in their case, the maximum equilibrium capacity is reached over a longer adsorption time. A different adsorption profile can be seen for the bioadsorbent AGW, which reaches equilibrium more slowly with a significantly slower adsorption rate. The CO_2_ adsorption profiles on biocarbons and biowastes for almost all samples are identical to those of the bioadsorbents derived from them. The exceptions are the CO_2_ adsorption profiles on the biocarbon CGCG, which differ from the adsorption profile for the bioadsorbent AGCG, and the CO_2_ adsorption profile on the biocarbon CPP, which differs from the adsorption profile for the bioadsorbent APP. CO_2_ adsorption on these two biocarbons and biowastes is slower than on the bioadsorbents obtained from them.

**Figure 15:**
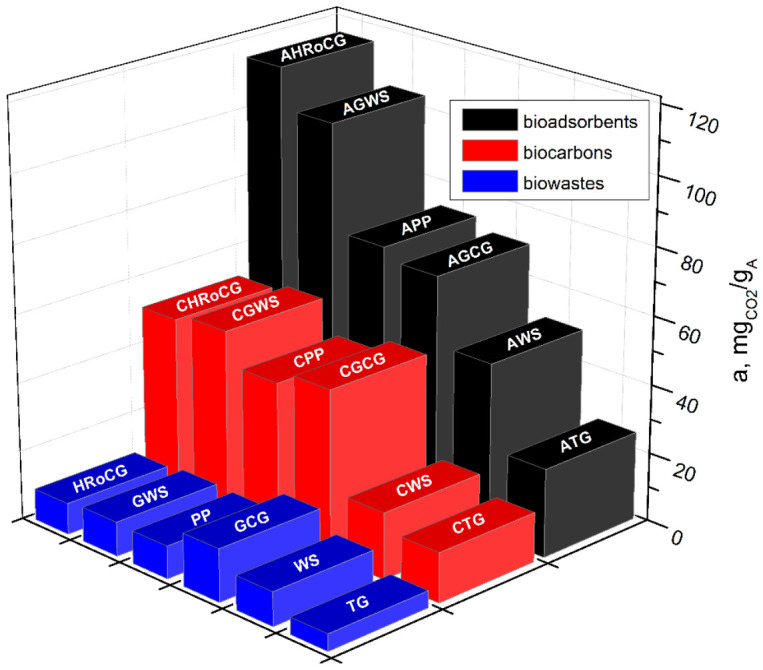
CO_2_ sorption capacity of bioadsorbents, biocarbon and biowastes.

**Figure 16:**
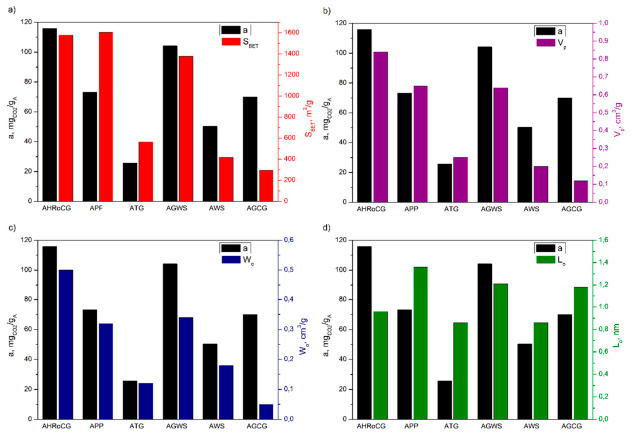
CO_2_ sorption capacity of different types of bioadsorbents versus (**a**) surface area, (**b**) pore volume, (**c**) micropore volume, and (**d**) pore diameter.

Figure 15 presents the CO_2_ sorption capacities for the six analyzed bioadsorbents and their precursors: biocarbons and biowastes. The results are arranged in descending order of CO_2_ sorption capacity for the obtained bioadsorbents. As shown in Figure 15, the highest CO_2_ sorption capacities were exhibited by the following bioadsorbents: coffee ground bioadsorbent (AHRoCG) with 115.8 mg_CO2_/g_A_, green walnut husk bioadsorbent (AGWS) with 104.1 mg_CO2_/g_A_, potato peel bioadsorbent (APP) with 73.2 mg_CO2_/g_A_, and green coffee ground bioadsorbent (AGCG) with 73.2 mg_CO2_/g_A_. The lowest CO_2_ sorption capacity was found for the tea ground bioadsorbent (ATG) with 25.8 mg_CO2_/g_A_. As shown in Figure 16, all bioadsorbents had higher CO_2_ sorption capacities compared to the biocarbons from which they were derived, confirming the effectiveness of the chemical modification of the biocarbons with KOH solution. Among the biocarbons, the highest CO_2_ sorption capacities were observed for the biocarbons from coffee grounds, green walnut husks, potato peels, and green coffee grounds: CHRoCG, CGWS, CPP, and CGCG.

These capacities were similar and ranged from 44.5 to 53.9 mg_CO2_/g_A_, with the highest being observed for the biocarbon from green walnut husks (CGWS). The lowest CO_2_ sorption capacity was exhibited by the biowastes, which were the initial precursors for the bioadsorbents. These materials adsorbed only small amounts of CO_2_, with the highest sorption capacity of 10 mg_CO2_/g_A_ found for the biowaste GCG (green walnut husks) and the lowest being 4.9 mg_CO2_/g_A_ for the biowaste TG (tea grounds). Considering the initial CO_2_ sorption capacity of the biowastes, the most significant increase in sorption capacity due to the processes of carbonization and KOH modification was observed for HRoCG and GWS. In the case of the HRoCG biowaste, the carbonization process resulted in more than a 5.5-fold increase in CO_2_ sorption capacity for the obtained biocarbon (CHRoCG), and taking the modification process into account, there was a 12.7-fold increase for the bioadsorbent (AHRoCG). Modification of the GWS biowaste led to a 5.2-fold increase in sorption capacity for the biocarbon (CGWS) and nearly a 10.4-fold increase for the bioadsorbent (AGWS). Biowastes like PP and GCG showed an increase in CO_2_ sorption capacity relative to biocarbons of 4.66 (CPP) and 3.14 (CGCG), while compared to bioadsorbents, the increases were 7.66 (APP) and 4.54 (AGCG). The remaining biowastes, WS and TG, exhibited the smallest increases in CO_2_ sorption capacity, i.e., 1.96 (CWS) and 2.93 (CTG) relative to the biocarbons and 5.07 and 5.21 relative to the bioadsorbents.

### 3.3. Effect of Porous Properties on CO_2_ Sorption Capacity of Biowaste, Biocarbon and Bioadsorbents

The proposed solid adsorbents in CO_2_ capture adsorption systems are physical adsorbents. They CO_2_ capture through intermolecular physical forces, which are weaker than those of chemical absorption, making it easier to regenerate the captured CO_2_ with a small amount of energy for reuse. The CO_2_ sorption capacity of physical adsorbents (such as activated carbons and zeolites) primarily depends on their porous properties, including pore structure and size. Figure 16 presents the CO_2_ sorption capacity of the studied bioadsorbents relative to their surface area, pore volume, micropore volume, and pore diameter. As shown in Figure 16a, three bioadsorbents with the highest CO_2_ sorption capacity—coffee ground bioadsorbent (AHRoCG, 115.8 mg_CO2_/g_A_), green walnut husk bioadsorbent (AGWS, 104.1 mg_CO2_/g_A_) and potato peel bioadsorbent (APP, 73.2 mg_CO2_/g_A_)—simultaneously had the highest specific surface area values among all the bioadsorbents: 1580 m^2^/g, 1376 m^2^/g, and 1604 m^2^/g for AHRoCG, AGWS, and APP, respectively.

**Figure 17:**
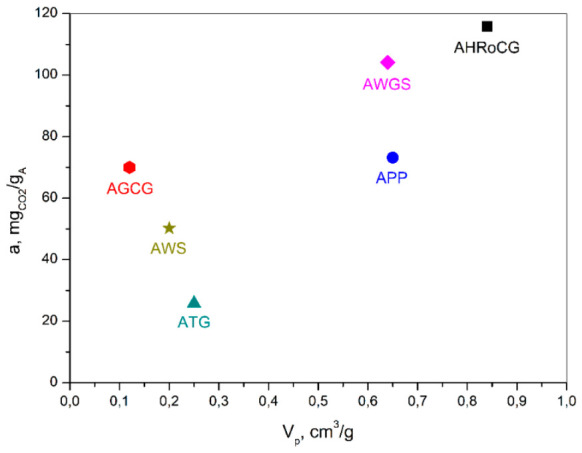
CO_2_ sorption capacity of different types of bioadsorbents versus total pore volume.

These bioadsorbents also exhibited the highest total pore volume values, amounting to 0.84 cm^3^/g, 0.64 cm^3^/g, and 0.65 cm^3^/g, for AHRoCG, AGWS, and APP, respectively (Figure 16b and Figure 17), as well as the highest micropore volume values of 0.5 cm^3^/g, 0.34 cm^3^/g, and 0.32 cm^3^/g, for AHRoCG, AGWS, and APP, respectively (Figure 16c). The highest CO_2_ sorption capacity, as shown in Figure 17, was observed for the bioadsorbents with the largest pore volume in their structure. Additionally, the coffee ground bioadsorbent (AHRoCG) also had one of the lowest average pore diameters among the tested bioadsorbents, measuring 0.96 nm (Figure 16d). The other two bioadsorbents with the highest CO_2_ sorption capacity, namely the green walnut husk bioadsorbent (AGWS) and the potato peel bioadsorbent (APP), had pores with a higher average pore diameter of 1.21 nm and 1.36 nm, respectively. A significant CO_2_ sorption capacity (70.02 mg_CO2_/g_A_) was shown by the green coffee ground bioadsorbent (AGCG), despite having the lowest specific surface area (293 m^2^/g) and the lowest total pore and micropore volumes, which were 0.12 cm^3^/g and 0.05 cm^3^/g, respectively. In contrast, bioadsorbents such as ATG and AWS, despite having pores with an average diameter below 1 nm (0.86 nm; 0.86 nm) and relatively high specific surface areas (564 m^2^/g; 416 m^2^/g), exhibited the lowest CO_2_ sorption capacities (25.8 mg_CO2_/g_A_; 50.2 mg_CO2_/g_A_). This was likely influenced by one of the lowest pore volumes among the tested bioadsorbents (0.25 cm^3^/g; 0.2 cm^3^/g) and micropore volumes (0.12 cm^3^/g; 0.18 cm^3^/g) in their structure. In summary, the modification of biochars with a KOH solution resulted in a significant increase in specific surface area and the creation of microporosity in most of the bioadsorbents, which enhanced their CO_2_ sorption efficiency. The initial biowaste materials, which lacked a developed porous structure, hardly sorbed CO_2_. The biochars obtained from their carbonization, which already had a developed specific surface area (from 0.2 to 289 m^2^/g) but only a limited pore and micropore volume, sorbed CO_2_ in the range of 14.5 to 53.9 mg_CO2_/g_A_ (Figure 15). It was only the modification of these biochars with the KOH solution that allowed the development of the porous structure, particularly microporosity, and thus increased CO_2_ sorption. The conducted studies confirmed that in CO_2_ capture using bioadsorbents, in addition to specific surface area, the presence of micropores with a diameter below 1 nm in their structure plays a crucial role.

This is due to the fact that as the pore radius decreases, the potential energy of adsorption increases. According to the literature [3,35,70,71], micropores with diameters ranging from 0.3 to 0.8 nm are favorable for effective CO_2_ adsorption, while the most effective pores are those with diameters below 0.5 nm [3]. The studied bioadsorbents, especially three of them with a significant proportion of micropores, demonstrated effective CO_2_ sorption. The diameters of CO_2_, O_2_, and N_2_ molecules present in exhaust gases are similar, measuring 0.33 nm, 0.346 nm, and 0.364 nm, respectively. When a CO_2_ molecule is near the nanopore wall, the C=O bond is immediately polarized due to the strong dispersion force between the oxygen atom and the carbon atom in the C=O bond [70]. The kinetic diameter of CO_2_ indicates that the ideal pore size should range from 0.5 to 0.6 nm. Pores within this size range allow CO_2_ molecules to interact with many parts of the porous adsorbent [71]. Such pore sizes were achieved in the studied bioadsorbents. This phenomenon can be explained by the fact that CO_2_ adsorption on porous carbon occurs according to the micropore filling mechanism, rather than monolayer adsorption. In practical exhaust gas conditions, the pore structure plays an important role in CO_2_ capture due to competitive adsorption with other components present in the exhaust gases. Therefore, the modification stage of the biochars obtained from the carbonization process is crucial in the production of CO_2_ bioadsorbents from biowastes in order to create a hierarchical pore structure with numerous narrow micropores, a large micropore volume, and a developed mesopore diffusion channel. Table 1 compares the CO_2_ adsorption capacities of the bioadsorbents studied in this work with those of selected bioadsorbents from the literature, considering the main parameters of their porous structures. The table includes biowastes obtained through chemical activation using potassium hydroxide solution. As shown in Table 1, the CO_2_ adsorption capacity of various bioadsorbents primarily depends on the parameters of their porous structure. The CO_2_ adsorption capacities at 25 °C for the bioadsorbents obtained in this study were comparable to those reported in the literature [22,23,31,38,39,40,50,51,61].

## 4. Conclusions

The use of bioadsorbents derived from household biowastes for CO_2_ capture can be an effective and environmentally friendly option for reducing greenhouse gas emissions. The bioadsorbents obtained through carbonization followed by chemical activation are carbon-rich materials characterized by developed porosity and high specific surface area. The conducted study demonstrated that the highest CO_2_ adsorption capacities among all investigated bioadsorbents were exhibited by three materials with the most advanced porous structures. The first of these, the bioadsorbent derived from roasted coffee grounds (AHRoCG) showed the most well-developed pore network. This bioadsorbent exhibited a high specific surface area (1580 m^2^/g), significant total pore volume (0.84 cm^3^/g), micropore volume (0.5 cm^3^/g), and an average pore diameter of 0.96 nm. These structural parameters directly translated into its CO_2_ adsorption capacity, which was the highest among all the tested materials, reaching 115.8 mg_CO2_/g_A_. Its high sorption performance was mainly attributed to the presence of narrow micropores, which possess a high adsorption potential. Similarly favorable structural properties were observed for the potato peel-derived bioadsorbent (APP—1604 m^2^/g; 0.65 cm^3^/g) and the bioadsorbent derived from green walnut shells (AGWS—1376 m^2^/g; 0.64 cm^3^/g). Their CO_2_ adsorption capacities reached 104.1 mg_CO2_/g_A_ and 73.2 mg_CO2_/g_A_, respectively, for AGWS and APP. The obtained results indicated that high CO_2_ uptake was achieved by those bioadsorbents which possessed not only a large specific surface area, but also a considerable pore volume—particularly in terms of micropores—and a favorable pore size distribution. The applied chemical activation of biochars using potassium hydroxide effectively enhanced their porous structure and promoted the formation of microporous networks. Therefore, the use of an appropriate activating agent for the biochar precursor is crucial, as it determines the effectiveness of CO_2_ physical adsorption. In conclusion, household biowastes represent a valuable raw material for producing sustainable, green, and efficient adsorbents for CO_2_ capture applications in the energy sector or other industrial branches.

## Figures and Tables

**Table 1:** Comparison of CO_2_ adsorption capacity of selected bioadsorbents derived from household biowastes.

Biowaste	Bioadsorbent	Pore Structure Parameters (S_BET_; V_p_; W_0_)	Sorption CO_2,_ mg_CO2_/g_A_ 25 °C, 1 Bar	Ref.
**coffee grounds**	CG800-1	1692; (-); (-)	195.0	[38]
	NCLK-3	840; (-); (-)	131.4	[40]
	CG 700 2-1	1624; (-); (-)	193.5	[39]
**coconut shell**	NC-650-4	1687; (-); (-)	210.0	[31]
	CN-600-3	1034; (-); (-)	162.0	[51]
	Cnut-3.5 h	1327; (-); (-)	170.0	[50]
**carrot peels**	CP	1379; 0.58; 0.51	185.0	[61]
**pomegranate peels**	PP-K-800	2144; 1.28; (-)	159.0	[22]
**waste tea**	WTAC	256.5; (-); (-)	87.4	[23]
**high roasted coffee grounds (HRoCG)**	AHRoCG	1580; 0.84; 0.5	115.8	This study
**potato peelings (PP)**	APP	1604; 0.65; 0.32	73.2	This study
**tea grounds (TG)**	ATG	564; 0.25; 0.12	25.8	This study
**green walnut shells (GWS)**	AGWS	1376; 0.64; 0.34	104.1	This study
**walnut shells (WS)**	AWS	416; 0.2; 0.18	50.2	This study
**green coffee grounds (GCG)**	AGCG	293; 0.12; 0.05	70.0	This study

## Data Availability

The original contributions presented in this study are included in the article. Further inquiries can be directed to the corresponding author.

## Nomenclature

AGCG	activated green coffee grounds (activated biocarbon)
AGWS	activated green walnut shells (activated biocarbon)
AHRoCG	activated high roasted coffee grounds (activated biocarbon)
APP	activated potato peelings (activated biocarbon)
ATG	activated tea grounds (activated biocarbon)
AWS	activated walnut shells (activated biocarbon)
CGCG	charred green coffee grounds (biocarbon)
CGWS	charred green walnut shells (biocarbon)
CHRoCG	charred high roasted coffee grounds (biocarbon)
CPP	charred potato peelings (biocarbon)
CTG	charred tea grounds (biocarbon)
CWS	charred walnut shells (biocarbon)
GCG	green coffee grounds (biowaste)
GWS	green walnut shells (biowaste)
HRoCG	high roasted coffee grounds (biowaste)
PP	potato peelings (biowaste)
TG	tea grounds (biowaste)
WS	walnut shells (biowaste)
a	CO_2_ sorption capacity, mg_CO2_/g_A_
L_o_	average pore diameter, nm
Q	theta angle, °
S_BET_	specific surface area, m^2^/g
t	time, min
T	temperature, °C
V_p_	total pore volume, cm^3^/g
W_o_	micropore volume, cm^3^/g
λ	wavenumber, cm^−1^
Va	volume adsorbed, cm^3^/g
D	pore diameter, nm
